# Hospital and patient influencing factors of treatment schemes given to type 2 diabetes mellitus inpatients in Inner Mongolia, China

**DOI:** 10.12688/f1000research.9095.1

**Published:** 2016-07-05

**Authors:** Nan Zhang, Edward McNeil, Sawitri Assanangkornchai, Yancun Fan

**Affiliations:** 1 School of Health Management, Inner Mongolia Medical University, Inner Mongolia, China; 2 Epidemiology Unit, Faculty of Medicine, Prince of Songkla University, Hat Yai, Songkla, Thailand

**Keywords:** Treatment scheme, healthcare disparities, T2DM, influence factors, hospital management

## Abstract

**Background**:

In clinical practice, the physician’s treatment decision making is influenced by many factors besides the patient’s clinical conditions and is the fundamental cause of healthcare inequity and discrimination in healthcare settings. Type 2 diabetes mellitus (T2DM) is a chronic disease with high prevalence, long average length of stay and high hospitalization rate. Although the treatment of T2DM is well guideline driven, there is a large body of evidence showing the existence of treatment disparities. More empirical studies from the provider side are needed to determine if non-clinical factors influence physician’s treatment choices.

**Objective**:

To determine the hospital and patient influencing factors of treatment schemes given to T2DM inpatients in Inner Mongolia, China.

**Methods**:

A cross-sectional, hospital-based survey using a cluster sampling technique was conducted in three tertiary hospitals and three county hospitals in Inner Mongolia, China. Treatment schemes were categorized as lifestyle management, oral therapy or insulin therapy according to the national guideline. Socio-demographic characteristics and variables related to severity of disease at the individual level and hospital level were collected. Weighted multinomial logistic regression models were used to determine influencing factors of treatment schemes.

**Results**:

Regardless of patients’ clinical conditions and health insurance types, both hospital and patient level variables were associated with treatment schemes. Males were more likely to be given oral therapy (RRR=1.72, 95% CI=1.06-2.81) and insulin therapy (RRR=1.94, 95% CI=1.29-2.91) compared to females who were given lifestyle management more frequently. Compared to the western region, hospitals in the central regions of Inner Mongolia were less likely to prescribe T2DM patients oral therapy (RRR = 0.18, 95% CI=0.05-0.61) and insulin therapy (RRR = 0.20, 95% CI=0.06-0.67) than lifestyle management. Compared with non-reformed tertiary hospitals, reformed tertiary hospitals and county hospitals were less likely to give T2DM patients oral therapy (RRR = 0.07 and 0.1 respectively) and insulin therapy (RRR = 0.11 and 0.17 respectively).

**Conclusion**:

Gender was the only socio-demographic factors associated with treatment scheme for T2DM patients. Hospitals from different regions have different T2DM treatment patterns. Implementation of reform was shown to be associated with controlling medication use for T2DM inpatients. Further studies are needed to investigate the causes of unreasonable treatment disparities so that policies can be generated accordingly.

## Introduction

Providing equitable and high quality healthcare is an essential objective of the health systems all over the world. However, variations in medical practices are observed across and within countries ^1, 2^ in the management of many diseases and clinical conditions ^3, 4^, including diagnosis, nursing care and treatment ^5^.

The differences in healthcare among different population groups are defined as healthcare disparities ^6^. More and more attention is now focused on health disparities that impair healthcare quality and overall health outcomes. It is estimated that $309 billion per year is lost due to the direct and indirect costs of health disparities in the United States of America ^7^.

Both clinical and non-clinical factors may lead to disparities in healthcare. From the view of healthcare equity, it is important to recognize the wide range of factors that contribute to disparities, especially non-clinical factors, such as socio-economic and healthcare system factors. Evidence from the population side suggests that a variety of factors are related to unequal health services received by people ^8– 10^. Although the influencing factors differ among countries and regions, demographic and socio-economic factors are proposed in many studies ^11, 12^. Since the clinical conditions of the people are very difficult to evaluate from most population-based surveys by self-reported health status, there are biases when comparisons of treatments are made between different population groups. A hospital-based study may overcome this limitation and provide physicians defined clinical and non-clinical patient information, so that they have more confidence in comparing the healthcare services they receive. Yet evidence from the provider side is limited, especially in low- and middle-income counties ^13^.

Inner Mongolia is a self-governed province located in the northern part of China. Compared to other parts of China, healthcare accessibility is inferior in Inner Mongolia because of the low population density, limited health resources and underdeveloped social-economic status. All of these characteristics may promote healthcare disparities.

Diabetes has become a large and rapidly-growing health problem. In 2011, it accounted for 8.2% of global all-cause mortality of people aged 20–79 ^14^. Type 2 diabetes mellitus (T2DM) is one of the leading chronic diseases in Inner Mongolia. The prevalence of diabetes in Inner Mongolia was 2.6% in 2008, according to the fourth National Health Service survey, an increase of 160% from 2003 ^15^. Besides having a high prevalence, diabetes is also associated with longer length of stay (LOS) in hospital and incurs a high medical expenditure. According to the data of Health Ministry of China in the year 2010 the average LOS of diabetes inpatients was 13.2 days, which was 3.2 days longer than the average LOS of all inpatients ^16^. Healthcare disparities may exist in treatment of T2DM inpatients since the disease is chronic and more expensive than other diseases.

Treatment of T2DM is well guideline-driven. The Chinese Diabetes Society (CDS) issued the Chinese Guideline for Treatment of T2DM and has revised it every 3 to 4 years ^17^. The International Diabetes Federation (IDF) and the American Diabetes Association (ADA) provide updated guidelines for T2DM treatment as well ^18^. Treatment schemes in all the T2DM treatment guidelines consistently include three components: lifestyle management, oral therapy and insulin therapy ^17, 19^. Since type 2 diabetes is a progressive disease, patients who have had a longer duration of disease are likely to have reduced beta-cell function and require more intensive therapy compared to patients with a more recently diagnosed disease ^20^.

A hospital-based study was implemented aiming to determine the hospital and patient influencing factors on treatment schemes given to T2DM inpatients in Inner Mongolia of China. Both clinical and non-clinical factors from hospital and patient perspectives were considered. The potential influencing factors considered in this study were categorized as socio-demographic (non-clinical factors in patient side), disease characteristics (clinical factors in hospital side) and hospital characteristics (non-clinical factors in hospital side).

## Methods

### Study design

A cross-sectional hospital-based study.

### Setting and participants

The population of this study comprised T2DM inpatients in Inner Mongolia of China. Inner Mongolia has three-tier hospitals. Tertiary hospitals located in urban area serve both urban and rural people. County hospitals are secondary hospitals serving rural people mostly. Community health institutions and township hospitals provide primary healthcare in urban and rural areas respectively. Moreover, there are Mongolian hospitals included in the hospital system of Inner Mongolia that mainly serve minority people and use totally different techniques and drugs from western medicine. Tertiary and county hospitals, as major providers of T2DM hospitalization care in Inner Mongolia, were chosen for this study. A multistage cluster sampling method was used to select the study sample. Inner Mongolia was geographically classified into three regions and the largest tertiary hospital and county hospital in each region were purposively selected. The eligible hospitals should have facilities to provide standard T2DM inpatient services as required by clinical guidelines. Only hospitals with good quality medical records and those where hospital charge information could be extracted from hospital information system were selected. Three tertiary hospitals and three county hospitals were finally chosen. All consecutive inpatients with a principle diagnosis of T2DM (ICD-10 codes: E11.2–E11.9) admitted into the sampled hospitals and discharged during the data collection period were recruited into this study. Those who stayed in hospital for less than 24 hours or could not communicate by themselves were excluded. Finally, a total of 771 eligible participants were recruited into this study.

Table 1 shows the characteristics of surveyed hospitals.

**Table 1.** Characteristics of the surveyed hospitals.

Surveyed hospital	Location	Hospital level	Beds*	Number of DM inpatients per year**	Sample size
1	Central	Tertiary	1891	1020	184
2	Western	Tertiary	2200	1000	180
3	Eastern	Tertiary	2000	954	170
4	Central	County	420	248	101
5	Western	County	382	150	59
6	Eastern	County	350	170	77

* Data were obtained from yearly statistical data of each hospital. ** Data were extracted from the hospital information system (HIS) of each hospital.

### Variables

***Outcome variables.*** The outcome variable of this study was treatment schemes of T2DM inpatients, which were classified into three categories: lifestyle management, oral therapy and insulin therapy, according to China’s treatment guideline for T2DM.

***Explanatory variables.*** Socio-demographic variables (age, gender, ethnicity, marital status, occupation, residence, education, yearly income and expenditure, insurance scheme), disease characteristics variables (duration of T2DM, Age-adjusted Charlson Comorbidity Index (ACCI) score, diagnosis, participation in treatment planning), and institutional variables (hospital level, location of hospital and hospital reform status) were considered as explanatory variables for treatment schemes of T2DM inpatients.

## Data collection and measurement

Patient interview data and medical records were collected and analyzed. Patient interviews were conducted one day before the patients were discharged using a questionnaire administered by well-trained research assistants. One week after the interview, the patients’ final medical records were reviewed by two researchers using a self-designed medical record review form. Both the patient interview questionnaire and the medical review forms were pre-tested in a pilot study for validation. A standard operating procedure was generated to standardize the data collection process.

Socio-demographic variables and two clinical variables (duration of T2DM and participation in treatment planning) were collected by patient interview, the other variables were extracted from the medical records.

Duration of T2DM was calculated by asking a patient the exact year when he/she was first diagnosed as T2DM by a clinician. ACCI scores were calculated automatically using an online calculator, with scores ranging from 0–37. The calculation was done by adding the weighted scores for 19 medical conditions. High scores are associated with a poorer prognosis ^21^. Diagnosis was coded as T2DM, T2DM with complications, T2DM with comorbidities, and T2DM with complications and comorbidities. Patient participation in treatment planning was determined by asking patients: “Did the doctors discuss your treatment plan with you before they made a decision?” Hospital reform status was confirmed with the local government. Only the hospital which had implemented the reform policies (including Zero mark-up on part of essential medicines and clinical pathway for T2DM) was defined as reformed hospital. Two of the surveyed county hospitals were assigned as pilot hospital of hospital reform under the framework of national medical reform, however, both of them were coded as non-reformed hospitals for having not started reform yet.

## Statistical methods

Data were presented as percentages for categorical variables or means for continuous variables. Multinomial logistic regression was used to explore the factors associated with treatment schemes. Weights were used to adjust the estimates due to cluster sampling. The strength of the association between factors and treatment scheme were presented as relative risk ratios (RRR) and 95% confidence intervals (95% CI). A P-value of less than 0.05 was considered as statistically significant. Data analysis was performed using the survey package of R software version 3.0.3 and Stata version 10.0. ****

## Ethical considerations

This study was approved by the Institutional Review Board of the Faculty of Medicine, Prince of Songkla University, Thailand. Permission was obtained from surveyed hospitals and written informed consent was obtained from each participant before enrollment in the study. No incentives or financial payments were provided for the interviewed patients. Personal identification was removed from the completed questionnaires and confidentiality was assured.

## Results

### Comparison of treatment schemes based on T2DM inpatients’ socio-demographic characteristics

Social-demographic characteristics of participants and their treatment schemes are shown in Table 2. 80% of T2DM inpatients were aged 50 years and above. 6.4% attained a bachelor degree study or above. Minority groups were rare (3.7%). More than half of the participants (59.5%) were living in urban areas. Although the national health insurance system (urban employee’s insurance scheme, urban resident’s insurance scheme and new rural cooperative medical system) covered most of the participants, 21.2% of the participants covered the cost of their hospitalization.

**Table 2.** Comparison of treatment schemes based on T2DM inpatients’ socio-demographic characteristics (%).

Variables	Total	Lifestyle management	Oral Therapy	Insulin therapy	P value
**Gender**					
Female	50.1	63.8	51.6	47.3	**0.03**
Male	49.9	36.2	48.4	52.7	
**Age group (years)**					
<50	20.0	13.8	16.4	21.8	0.05
50–59	28.2	17.8	29.4	29.8	
60–69	30.4	47.2	33.9	26.7	
≥70	21.4	21.2	20.2	21.7	
**Education**					
Illiterate	20.5	32.0	16.5	19.3	0.32
Primary school	18.1	14.9	24.8	17.2	
High school	54.9	48.0	51.9	56.8	
Bachelor or above	6.5	5.2	6.8	6.7	
**Ethnicity**					
Han	96.3	97.7	94.4	96.5	0.53
Minority	3.7	2.3	5.6	3.5	
**Occupation**					
Unemployed	55.1	58.0	57.4	54.2	0.71
Retired	21.2	24.7	21.2	20.6	
Employed	23.7	17.3	21.4	25.3	
**Marital status**					
Married	88.8	87.4	91.8	88.4	0.50
Single/separated/widowed/divorced	11.2	12.4	8.2	11.6	
**Household yearly income (Yuan)**					
<20000	29.3	32.1	35.0	27.7	0.50
20000–40000	23.9	31.6	14.6	24.4	
40000–60000	18.5	12.5	19.6	19.2	
>60000	28.3	23.7	30.8	28.6	
**Household yearly expenditure (Yuan)**					
<20000	34.7	0.44	39.1	32.1	0.50
20000–40000	32.0	23.7	26.2	34.7	
40000–60000	16.4	19.3	19.9	15.2	
>60000	16.8	12.5	14.7	18.0	
**Location**					
Rural areas	40.5	49.6	45.8	37.8	0.37
Urban areas	59.5	50.4	54.2	62.2	
**Payers**					
Uninsured	21.2	17.9	11.5	23.8	0.49
UEIS	33.4	28.9	33.1	34.2	
URIS	13.2	15.0	12.1	13.1	
NRCMS	31.1	36.2	42.8	27.7	
Other insurance	1.2	2.1	0.6	1.1	

UEIS: Urban employee’s insurance scheme URIS: Urban resident’s insurance scheme NRCMS: New rural cooperative medical system

Treatment schemes significantly differed between genders. Females were more likely to be given lifestyle management and oral therapy, whereas males were more likely to be given insulin therapy. Lifestyle management and oral therapy were mostly given to those aged 60–69, those aged 50–59 were more likely to be given insulin therapy, however, the p-value was just on the cut point of statistical significance.

### Comparison of treatment schemes based on T2DM inpatients’ clinical characteristics

Table 3 shows treatment schemes in relation to T2DM inpatients’ clinical characteristics. Diagnosis was significantly associated with treatment schemes. Those with comorbidities were more likely to be given lifestyle management and oral therapy than those with other clinical types, whereas those with complications and comorbidities were more likely to be given insulin therapy. The average duration of T2DM was 7.3 years (SE=0.5) and the mean ACCI score was 5.0 (SE=0.5). Physicians discussed with most of T2DM inpatients (85%) when they planned the treatment scheme for them.

**Table 3.** Comparison of treatment schemes based on T2DM inpatients’ clinical characteristics (%).

Variables	Total	Lifestyle management	Oral Therapy	Insulin therapy	P value
**Diagnosis**						
Simple T2DM	2.7	0.0	1.4	3.5	**0.01**
T2DM + complication	3.9	2.1	3.2	4.3	
T2DM + comorbidity	54.0	86.5	65.7	45.8	
T2DM + complication + comorbidity	39.4	11.4	29.6	46.3	
**Duration of T2DM (years)**						
Mean(SD)	7.3(0.5)	6.6(0.7)	5.6(0.9)	7.8(0.4)	0.31
**ACCI scores**						
Mean(SD)	5.0(0.5)	4.9(0.2)	5.0(0.4)	5.1(0.6)	0.75
**Participation in treatment planning**						
Yes	85.0	86.5	84.6	84.9	0.94
No	15.0	13.5	15.4	15.1	

### Comparison of treatment schemes based on hospital characteristics

Table 4 shows the T2DM inpatient treatment schemes in different hospitals. Treatment schemes for T2DM inpatients differed in hospitals located in different regions. Lifestyle management and oral therapy were given more in the Eastern region, whereas insulin therapy was given more in the Western and central regions. Since there were no county hospitals served as pilot hospital for medical reform when we conducted this survey, hospital level and reform status were combined into one category variable to be analyzed. In the tertiary hospitals, which were not involved in medical reform, insulin therapy was common, whereas in the tertiary hospital with reform, oral therapy was used most. County hospital gave T2DM inpatients lifestyle management more frequently than medication. However, all the differences above were not statistically significant.

**Table 4.** Comparison of T2DM inpatient treatment schemes by hospital characteristics (%).

	Total	Lifestyle management	Oral therapy	Insulin therapy	P value
**Hospital location**						
Western	33.3	19.0	22.0	38.2	0.07
Eastern	33.3	46.5	59.1	25.6	
Central	33.3	34.5	18.8	36.2	
**Hospital reform status**						
Tertiary hospitals without reform	16.3	3.5	12.2	19.4	0.47
Tertiary hospitals with reform	32.6	33.5	36.5	31.6	
County hospitals	51.1	63.0	51.3	49.0	

### Hospital and patient factors associated with treatment schemes given to T2DM inpatients

Table 5 summarizes the results of a multivariate analysis for hospital and patient factors associated with treatment schemes given to T2DM inpatients. After controlling for patients’ clinical characteristics and payers, both hospital and patient level variables were associated with treatment schemes. Males were more likely to be given oral therapy (RRR = 1.72) and insulin therapy (RRR = 1.94) than females. Compared to other clinical types, those with comorbidities were less likely to be given insulin therapy (RRR = 0.16). Compared with the Western region, hospitals in central regions of Inner Mongolia were less likely to give T2DM inpatients oral therapy (RRR = 0.18) and insulin therapy (RRR = 0.20) than lifestyle management. Compared with tertiary hospitals without doing any healthcare reform, tertiary hospitals with reform and county hospitals were less likely to give T2DM inpatients oral therapy (RRR = 0.07 and 0.1 respectively) and insulin therapy (RRR = 0.11 and 0.17 respectively).

**Table 5.** Hospital and patient factors associated with treatment schemes given to T2DM inpatients.

	Oral therapy			Insulin therapy		
	RRR	95% CI	P	RRR	95% CI	P
Individual level variables						
**Gender**(Ref.=Female)						
Male	1.72	1.06–2.81	**0.04**	1.94	1.29–2.91	**0.01**
**Diagnosis**(Ref.= Other clinical types)						
T2DM with comorbidity	0.30	0.08–1.13	0.07	0.16	0.06–0.44	**0.01**
**Duration of T2DM**	0.98	0.94–1.03	0.37	1.03	0.99–1.08	0.13
**ACCI score**	0.95	0.81–1.12	0.49	0.89	0.72–1.09	0.19
**Payers**(Ref.= uninsured)						
UEIS	0.81	0.13–5.20	0.79	0.58	0.07–5.15	0.55
URIS	0.78	0.07–8.28	0.80	0.95	0.04–20.26	0.97
NRCMS	1.17	0.21–6.48	0.83	0.62	0.12–3.26	0.49
Other insurance	0.20	0.01–6.69	0.30	0.31	0.01–15.8	0.48
**Hospital level variables**						
**Hospital location**(Ref.= Western region)	
Hospital located in eastern region	1.39	0.41–4.71	0.52	0.37	0.12–1.09	0.06
Hospital located in central region	0.18	0.05–0.61	**0.02**	0.20	0.06–0.67	**0.02**
**Hospital reform status**(Ref.= tertiary hospital without reform)						
Tertiary hospital with reform	0.07	0.02–0.31	**0.01**	0.11	0.02–0.50	**0.01**
County hospital	0.10	0.02–0.58	**0.02**	0.17	0.03–0.94	**0.04**

UEIS: Urban employee’s insurance scheme URIS: Urban resident’s insurance scheme NRCMS: New rural cooperative medical system RRR: relative risk ratio. Reference outcome group = lifestyle management CI: confidence interval

Raw data of hospital and patient influencing factors on treatment schemes given for type 2 diabetes mellitus in patients in Inner Mongolia The raw data of influencing factors on treatment schemes are provided. Click here for additional data file. Copyright: © 2016 Zhang N et al. 2016 Data associated with the article are available under the terms of the Creative Commons Zero "No rights reserved" data waiver (CC0 1.0 Public domain dedication).

## Discussion

After controlling for clinical conditions such as duration of disease, ACCI score and diagnosis, the hospital level factor stood out of all the potential explanatory variables. Hospital location, reform status and hospital level were associated with treatment scheme for T2DM inpatients significantly, while gender was the only demographic variable related.

Compared with the Western region, T2DM patients treated in the central regions of Inner Mongolia were more likely to be given lifestyle management than medication. As one of the major causes for healthcare disparities, geographic variations in treatment pattern of T2DM have been reported in other studies ^22^. However, the underlying reasons of the variations were not always the same ^23^. There might be two possible explanations for regional variations in this study. First, the Western region in Inner Mongolia has a lower population density compared with the central region (23 person/km ^2^ vs 174 person/km ^2^). Since a general practice system has not been fully set up in China, especially in Inner Mongolia, inferior accessibility to healthcare may reduce the healthcare seeking behavior of T2DM patients, which will cause them to lose the opportunity for early treatment. Second, hospitals in the same region have more chance to have professional and academic exchanges and learn from each other under the circumstance of lacking clinical pathway management. More evidences are needed to get the precise information on the factors contributing to the variations so that policies could be designed accordingly.

In this study we show that implementation of reform was associated with controlling medication use for T2DM patients. Reformed tertiary hospitals were less likely to give T2DM patients medication treatment compared to tertiary hospitals without any reform. The public hospital reform in China under the national healthcare reform framework started from tertiary hospitals ^24^. In the first stage of the reform, enhancing internal management was carried out by many hospitals to improve the quality of care. Zero mark-up on part of essential medicines (A zero-profit drug policy introduced by China government from the year 2009 to remove the mark-up for medicines sale in hospitals. The major objective of this policy was to reduce the incentive for providers to prescribe unnecessary drugs ^25^.), and clinical pathway for T2DM were implemented in the reformed hospitals of this study. Although the hospitals had set out to implement the reform for less than three years when this survey was conducted, a series of policies was generalized and experimented by the hospitals to match the requirement of the government. Some studies report that the zero mark-up policy can decrease drug prescriptions and reduce total expense for both outpatient and inpatient services ^26, 27^. Clinical pathway has been shown to reduce the variability in clinical practices ^28^. However, further studies are needed to confirm the causation between the reform and the change in physicians’ clinical practices.

Among non-clinical characteristics, major gender differences in the treatment schemes given to T2DM patients were observed in this study. Males were more likely to be given oral therapy and insulin therapy than females who were mostly given lifestyle management. Many studies have revealed the fact that females have increased adherence to preventive practices ^29^, and males have lower obedience to diet control and exercise ^30^. However, gender differences in treatment protocols lead to unequal treatment and it should be avoided as much as possible. One way this can be done is by incorporating patient preferences into the treatment schemes. Systematic reviews done on the patient preferences for non-insulin diabetes medications during 2007–2012 ^31, 32^ have shown the importance of incorporating patient preferences into treatment decisions. These reviews showed that the key attributes of diabetes medication associated with patient preferences include treatment benefits ( *e.g.* glycemic control and weight loss/control), treatment burden ( *e.g.* administration, frequency, and cost), and side effects ( *e.g.* weight gain, gastrointestinal effects, and hypoglycemia) ^31^. Therefore, gender differences in the treatment schemes can be allowed as long as they accommodate the patient’s preferences and treatment efficacy is not compromised.

A number of studies have found evidence about health disparities in different ethnic groups ^33, 34^. However, ethnicity was not significantly associated with treatment schemes in this study. If we look at the distribution of ethnicity among T2DM inpatients, it was quite different from that of the whole population of Inner Mongolia. Our findings showed that minority groups with T2DM were rare (3.7%) across the six hospitals studied. This proportion was much lower than in the whole population of Inner Mongolia (21%). This could be because minorities prefer traditional medicine to Western medicine. However, further studies are needed to obtain information about accessibility and utilization of healthcare for minority groups with T2DM in Inner Mongolia. Where ethnic and racial disparities exist in the treatment of diabetes patients, it is evident that policy responses for addressing minority groups are needed.

Education level was low among T2DM patients in this study. Only 6.5% of T2DM patients had attained a bachelor degree or above, which was lower than the whole population (10.3%). Education has been found to be associated with all aspects of T2DM treatment including prevention, outpatient care, inpatient care, and rehabilitation ^29, 35^. However, other studies have put forward evidence that the educational level of patients had no association with untreated diabetes ^36^ or with the prevalence of diabetes ^37^. Although the possibilities for disparities in treatment types may exist by level of education, this study did not find any association between education level and the type of treatment schemes that was given to the patients.

This study also found that although the national health insurance system covered most (77.6%) of the participants, 21.2% of the participants had no insurance. In contrast, among all adults with diabetes in the USA, 92.0% had some form of health insurance ^38^. Physicians usually have different personal attitudes toward insured and uninsured patients. This was demonstrated in an analysis of patients who were insured which showed that they had a higher number of prescribed medications, and a higher total price of prescription than those who paid cash only ^39^. It has been found that even insurers have major differences in attitude with regard to diabetes. Some patients who had declared their disease status to the insurers were refused acceptance for insurance and for some, their premiums were increased ^40^. These evidences prove the importance of state mandated insurance programs, especially for diseases such as T2DM which has a long treatment period.

## Strengths and limitations

This was a hospital-based study using combined methods of patient interview and hospital record review to obtain clinical and non-clinical data, which can eliminate the recall bias on health service experience inherent in population based surveys. However, several limitations in this study should be acknowledged. First, it was a hospital-based study, therefore the findings cannot be generalized to the whole population of China, or T2DM patients. Second, due to the cross-sectional nature of the study, causality of certain risk factors and the study outcome cannot be established. Third, we did not measure all potential patient risk factors, such as hemoglobin A1C values, which were not available. As such, duration of T2DM, diagnosis and ACCI score were used to estimate severity of disease, which are known to be less precise. Fourth, physicians’ characteristics (such as age, how long they were certified, *etc*.) were not available, which would be a factor in determining treatment and adherence to the national guidelines.

## Conclusion

In conclusion, this study did not show any socio-demographic factors except gender influencing the treatment patterns for T2DM inpatients, independently of their clinical condition. Hospitals from different regions have different treatment patterns, which tends to increase the healthcare disparities and should be eliminated by exploring further policy strategies. Implementation of reform was shown to be associated with controlling medication use for T2DM inpatients. However, the causation between the reform and changing of physicians’ clinical practices should be explored in further studies.

## Data availability

The data referenced by this article are under copyright with the following copyright statement: Copyright: © 2016 Zhang N et al.

Data associated with the article are available under the terms of the Creative Commons Zero "No rights reserved" data waiver (CC0 1.0 Public domain dedication). 

F1000Research: Dataset 1. Raw data of hospital and patient influencing factors on treatment schemes given for type 2 diabetes mellitus in patients in Inner Mongolia, 10.5256/f1000research.9095.d127825 ^41^
