# Supplementary material for: Hospital and patient influencing factors of treatment schemes given to type 2 diabetes mellitus inpatients in Inner Mongolia, China
Source: F1000Res. 2016 Jul 5;5:1577. [Version 1] doi: 10.12688/f1000research.9095.1 (PMC4943291; doi:10.12688/f1000research.9095.1)
Supplement: Peer review history

# Peer review: Referee response for version 1

**DOI:** 10.5256/f1000research.9789.r14809

**Published:** 2016-07-12

**Authors:** Jian Wang

**Affiliations:** 1 Department of Social Medicine and Administration, Shandong University, Jinan, China

This is a policy-relevant article to study hospital and patient factors influencing T2DM treatment in Mongolia. The rapid increasing of T2DM gives a pressure to the health system in Inner Mongolia. The authors are interested in exploring health care disparities by conducting a cross-section hospital survey in 6 hospitals in 3 regions. I would approve the article. However, there may be minor revisions if the authors accept the following comments.

1. In the limitation section, it would be better to explain if there any recall bias when using data from patient interview? If so, please state in limitation part.
2. Give a few words to demonstrate “a series of policies was generalized and experimented by the hospitals” at the current health reform.

I have read this submission. I believe that I have an appropriate level of expertise to confirm that it is of an acceptable scientific standard.

# Peer review: Referee response for version 1

**DOI:** 10.5256/f1000research.9789.r14808

**Published:** 2016-07-11

**Authors:** Stephen J Nicholas

**Affiliations:** 1 Newcastle Business School, The University of Newcastle, Newcastle, NSW, Australia

The paper briefly sets out on page 3 the nature of equity issues in hospital health care and the problems of measuring health care disparities, especially from the clinical side. Using a transitional and developing region of China, Inner Mongolia, the paper focuses on the widely accepted practice of measuring disparities from the population side (socio-economic characteristics) factors. Importantly, the paper also focuses on different types of hospital and also measures clinical treatment differences for diabetes (T2DM) treatments. The choice of disease, T2DM, is justified as the research focus. The authors should be commended for their access to treatment records.

 On page 3, the authors might add a couple of sentences on tertiary versus county hospitals, especially given the differences in bed-size. Also, the choice of hospital depended on the quality of the records, and a sentence on differences (if any) in tertiary versus county hospital records would be useful.

 In terms of the explanatory variables (page 3), the authors might briefly define insurance scheme, hospital reform status and diagnosis in this section (although these are (partly) covered in the section on ‘Data collection and measurement’).

 On page 8, the argument should make clear that type of insurance and ‘no insurance’ did not have an impact on treatment. The discussion contrasts the Inner Mongolia case of no differences in treat by insurance/’no insurance’ with other countries, but does not emphasize that there are no differences in the Inner Mongolia data between insurance/'no-insurance'. This requires only one sentence.

I have read this submission. I believe that I have an appropriate level of expertise to confirm that it is of an acceptable scientific standard.
